# Distinct roles for the IIId2 sub-domain in pestivirus and picornavirus internal ribosome entry sites

**DOI:** 10.1093/nar/gkx991

**Published:** 2017-10-24

**Authors:** Margaret M. Willcocks, Salmah Zaini, Nathalie Chamond, Nathalie Ulryck, Delphine Allouche, Noemie Rajagopalan, Nana A. Davids, Ulrik Fahnøe, Johanne Hadsbjerg, Thomas Bruun Rasmussen, Lisa O. Roberts, Bruno Sargueil, Graham J. Belsham, Nicolas Locker

**Affiliations:** 1 Faculty of Health and Medical Sciences, School of Biosciences and Medicine, University of Surrey, Guildford, UK; 2 Faculté des Sciences Pharmaceutiques et Biologiques, UMR8015, Université Paris Descartes, Paris, France; 3 DTU National Veterinary Institute, Technical University of Denmark, Lindholm, DK-4771 Kalvehave, Denmark; 4 School of Molecular and Cellular Biology, Faculty of Biological Sciences, University of Leeds, Leeds, UK

## Abstract

Viral internal ribosomes entry site (IRES) elements coordinate the recruitment of the host translation machinery to direct the initiation of viral protein synthesis. Within hepatitis C virus (HCV)-like IRES elements, the sub-domain IIId(1) is crucial for recruiting the 40S ribosomal subunit. However, some HCV-like IRES elements possess an additional sub-domain, termed IIId2, whose function remains unclear. Herein, we show that IIId2 sub-domains from divergent viruses have different functions. The IIId2 sub-domain present in Seneca valley virus (SVV), a picornavirus, is dispensable for IRES activity, while the IIId2 sub-domains of two pestiviruses, classical swine fever virus (CSFV) and border disease virus (BDV), are required for 80S ribosomes assembly and IRES activity. Unlike in SVV, the deletion of IIId2 from the CSFV and BDV IRES elements impairs initiation of translation by inhibiting the assembly of 80S ribosomes. Consequently, this negatively affects the replication of CSFV and BDV. Finally, we show that the SVV IIId2 sub-domain is required for efficient viral RNA synthesis and growth of SVV, but not for IRES function. This study sheds light on the molecular evolution of viruses by clearly demonstrating that conserved RNA structures, within distantly related RNA viruses, have acquired different roles in the virus life cycles.

## INTRODUCTION

The initiation of protein synthesis on the majority of cellular mRNAs is achieved via a cap-dependent scanning mechanism, reviewed in (1). The 5′-cap structure of cellular mRNAs is first recognized by the eukaryotic initiation factor complex 4F (eIF4F), comprising eIF4E, eIF4A and eIF4G (1). Then, eIF4F recruits onto the mRNA a 43S pre-initiation complex consisting of a 40S ribosomal subunit, the ternary complex eIF2-GTP-^Met^tRNA_i_, eIF3, eIF1 and eIF1A, thereby priming the canonical scanning mechanism (1). Upon base pairing of the start codon with the anticodon loop of the ^Met^tRNA_i_, a 48S complex is formed, triggering eIF5-mediated GTP hydrolysis by eIF2 and the joining of the 60S subunit promoted by eIF1A and a second GTP hydrolysis step driven by eIF5B. Bound eIFs then dissociate from the ribosomal subunits terminating the initiation stage of translation with the assembly of an elongation competent 80S ribosome. In contrast, initiation of translation on certain viral RNAs (e.g. from picornaviruses and pestiviruses) and some cellular mRNAs occurs via a cap-independent mechanism in which IRES elements within the RNA recruit the ribosome internally, bypassing the need for some of the eIFs and a 5′-cap recognition event (2). Internal entry of ribosomes can allow the preferential translation of the viral mRNAs compared to the bulk of cellular mRNAs thus providing an advantage for viral protein synthesis, as both viruses and the host compete for the translation machinery and viruses can trigger a shut-off of host cell translation. IRES elements have been discovered in the genomes of a number of RNA viruses, for example within the 5′-untranslated region (UTR) of the *Picornaviridae* and some members of the *Flaviviridae*. Many studies suggest that structured RNA domains within these elements specifically interact with eIFs or even directly with the ribosome to mediate the initiation of translation (3–5). Based on shared sequences, structural motifs and common interactions with the translation machinery IRES elements have been grouped into different classes (6–8), although not all clearly fall into these classes (i.e (9–12)).

A key feature of IRES-mediated translation, shared by many but not all IRES elements, is the direct interaction of the IRES with the 40S small ribosomal subunit (8,13). This interaction is critical for the initiation of translation on the group III IRES elements, the best studied examples of which are present in the RNA genomes of hepatitis C virus (HCV) and classical swine fever virus (CSFV). The HCV and CSFV IRES elements share most of the same RNA structure scaffold, comprising two major complex domains, termed II and III (14).

The basal region of domain III binds the back of the 40S ribosomal subunit with high affinity (15). It is responsible for positioning the start codon, located in the HCV domain IV, in the ribosomal decoding groove through several high affinity direct interactions mediated by sub-domains IIIa, IIIc, IIId, IIIe and IIIf (15–18). Recent biochemical and structural studies have highlighted the importance of the direct base-pairing between group III IRESes and the 18S rRNA extension segment 7 (ES7) (16,18–21). The apical part of domain III, termed IIIabc, and the four-way junction at its base, mediate the binding of eIF3 and the ternary complex to form 48S complexes (22–24). Although it contacts the 40S ribosomal subunit, the L-shaped elongated domain II is not required for 48S complex formation but drives the assembly of 80S ribosomes (25).

This mechanism of ribosome recruitment is shared by several IRES elements and is used by the HCV-like IRES elements found in the *Flaviviridae* but also in some of the *Picornaviridae* (26–30). Despite their functional similarities, some differences exist between the HCV and CSFV IRES elements. Of particular note, the CSFV IRES contains an additional sub-domain, termed IIId2, adjacent to the ribosome binding site IIId1 (31). This feature is shared with other pestivirus IRES elements such as that present in border disease virus (BDV) or bovine viral diarrhoea virus (BVDV) and also in certain picornavirus IRES elements such as in Seneca Valley virus (SVV). This characteristic differentiates the CSFV-like IRES elements from the HCV IRES. We previously reported that the SVV IRES is structurally and functionally related to the CSFV IRES (28). The deletion of the conserved SVV IIId2 sub-domain had no apparent effect either on SVV IRES activity, or on 40S and eIF3 binding. In the 40S-bound CSFV IRES structure, sub-domain IIId2 extends away from the surface of the 40S subunit, while foot-printing studies suggested this sub-domain is protected from RNAse V1 cleavage by the 40S subunit (16,17). Using different CSFV-like IRES elements we have now undertaken to define the role of sub-domain IIId2 in virus infectivity. First, we have shown that a sub-domain IIId2 is actually formed in the pestivirus BDV and CSFV IRES elements. Then, using bicistronic reporter assays, we demonstrated that deletion of sub-domain IIId2 impairs both BDV and CSFV IRES activity, while it was previously shown to have no impact on SVV IRES function. We further demonstrate that the IIId2 sub-domains of pestivirus IRES elements are required for the 80S ribosome assembly but not for the formation of 48S complexes. We show that deletion of sub-domain IIId2 impairs the infectivity of BDV and CSFV but also to a lesser extent that of SVV. Therefore, while the sub-domain IIId2 found in pestivirus IRES elements is important for both IRES activity and infectivity, in the IRES of the picornavirus SVV it plays a role in RNA replication but not in IRES activity. Overall, this suggests that these viruses have acquired conserved domains, presumably through recombination events, and that these may have evolved divergent functions in picornaviruses and pestiviruses.

## MATERIALS AND METHODS

### Cell lines

Human embryo kidney 293 cells were grown at 37°C in an atmosphere with 5% CO_2_ in Dulbecco's MEM (1 g/l glucose) supplemented with 10% fetal calf serum (FCS), 2% non-essential amino acids and 4 mM l-glutamine. Similarly, PK15 (porcine kidney) and SFT-R (sheep foetal thymoid) cells were grown in Dulbecco's MEM supplemented with 5% FCS, and BHK21 cells were grown in Dulbecco's MEM supplemented with 10% FCS and 10% tryptose phosphate broth.

### Plasmids

Full-length cDNAs corresponding to the coding sequences of CSFV and BDV genomes, cloned within the pBeloBAC11 vector, and termed pBeloPader10 (31–33) and pBeloGif09.9.2 have been described elsewhere ((33); Fahnøe *et al.*, in preparation). Modifications to the viral cDNAs were performed using targeted modification, as described in (34,35), resulting in the following IIId2 mutants: CSFV ΔIIId2 (deletion of AUCACACCAUGUGAU); CSFV loopIIId2 (formation of AUCACAGGUUGUGAU); CSFV stemIIId2 (formation of AAGAGACCAUGAGAU); BDV ΔIIId2 (deletion of AACGUCCUAGUGGCGUU); BDV loopIIId2 (formation of AACGUCCAUCUGGCGUU); BDV stemIIId2 (formation of AAGCGGCUAGUGGCGUU) (see also Figure 2). All sequences were verified by full genome sequencing using an Ion PGM (Life Technologies). SVV IRES sequences containing either mutations in the IIId2 sub-domain stem (stemIIId2), or deletion of the loop (loopIIId2) or of the entire sub-domain (ΔIIId2) (prepared in pGEM-CAT/LUC vector as described in (28)) were transferred into the full-length infectious SVV construct pNTX-09 (described in (36)) in the following manner. Firstly, using a site-directed mutagenesis kit (QuikChange; Stratagene) according to the manufacturer's instructions, a unique *Age I* restriction site in the virus was removed by a silent mutation (T to A) at nucleotide 6695 in the virus sequence (accession number DQ641257) using the following primer pair Mut Age I: 5′-CATCAAGATTACCGGAGGCCTCCCCTCCGG-3′ and 5′-CCGGAGGGGAGGCCTCCGGTAATCTTGATG-3′. a new recognition site for the same restriction enzyme was created by the same method, again by a silent mutation (A to T) at nucleotide 726 using primer pair Insert Age I: 5′-CACCTTTGAAGATGTAACCGGTACAAAAGTCAAGATCG-3′ and 5′- CGATCTTGACTTTTGTACCGGTTACATCTTCAAAGGTG-3′. Both mutations were verified by sequence determination. The resulting plasmid (pNTX-09-Age) was digested with *Hpa I* and *Age I* to remove bases 59 to 726. The mutated IRES cDNA sequences were amplified from the pGEM-CAT/SVV IRES/LUC constructs by PCR using primers SVV For2 (5′-ATATGGATCCTTTGAAATGGGGGGCTG-3′) and Insert Age Rev (5′-CGATCTTGACTTTTGTACCGGTTACATCTTCAAAGGTG-3′), the PCR products were digested with *Hpa I* and *Age I*, gel purified and ligated into the similarly digested pNTX-09-Age plasmid. Constructs were verified by restriction enzyme digestion and sequence determination of the entire SVV IRES region.

To generate the BDV and CSFV bicistronic constructs, fragments encoding the different IRES cDNA sequences were amplified by PCR from the full-length infectious viruses described before. The IRES fragments were cloned using standard molecular cloning into the pGEM-CAT/LUC plasmid described previously which encodes chloramphenicol acetyltransferase (CAT) and firefly luciferase (fLUC) under the control of a T7 promoter (37). The constructs were confirmed by restriction enzyme digestion, PCR and sequencing.

### Transfections and luciferase assays

Transient DNA transfections of BHK cells were performed in 35-mm dishes as described previously (37). 2 μg of each plasmid was transfected into cells previously infected with a recombinant vaccinia virus vTF7–3 (38), which expresses the T7 RNA polymerase, using 8 μl of Lipofectin (Invitrogen) and 192 μl of Optimem (Life Technologies). Cell lysates were prepared 20h post-transfection, fLUC expression was quantified using a Firefly Luciferase Assay kit (Promega) and detection in a luminometer (Labtech) and CAT protein measured by an enzyme-linked immunosorbent assay (ELISA) (Roche) and quantified in a plate reader (BioTek Instruments).

### Filter binding assays

The filter binding assays were performed as described (28). CSFV and BDV 5′ UTR RNAs were transcribed *in vitro* in the presence α-^32^P-UTP (3000 mCi/mmol) using T7 RNA polymerase and purified by size exclusion chromatography. The 40S ribosomal subunits and the purified initiation factor eIF3 were prepared from HeLa cells following established procedures (39). Radiolabeled RNA (50 fmol) was incubated with serial dilutions of either the 40S subunit or eIF3 in binding buffer (20 mM Tris, 100 mM KCl, 2 mM DTT and 2 mM MgCl_2_ [pH 7.6]) and incubated at 37°C for 15 min before performing filter binding assays using two filters as described (9). Bound RNA was quantified using a Typhoon FLA7000 phosphorimager (GE Healthcare).

### RNA structure determination

The secondary structure of the CSFV and BDV IRES IIId2 sub-domains was probed using dimethyl sulphate (DMS), *N*-cyclohexyl-*N*’-[*N*-methylmorpholinoethyl]-carbodiimid-4-toluenesulphate (CMCT) and RNAse V1 and analysed using reverse transcription as described (28,40,41). *In vitro* synthesis and purification of RNA from constructs containing the CSFV 5′ UTR (52–427) or the BDV 5′ UTR (59–430) in pGEM 3Z vector (Promega) was carried out using the MegaScript T7 kit (Ambion) according to the manufacturer's instructions. Subsequently, 2 pmol of each RNA was resuspended in 20 μl of either 20 mM HEPES, 100 mM K acetate, 200 mM KCl, 2.5 mM MgCl_2_ and 1 mM dithiothreitol (DTT) (pH 7.5) for DMS, 50 mM borate–NaOH and 1 mM EDTA (pH 8.0) for CMCT, or 20 mM Tris, 5 mM MgCl_2_ and 100 mM KCl (pH 7.5) for RNase V1, denatured for 1 min at 95°C and cooled on ice. DMS (0.395 M), CMCT (2, 4 or 10 mg/ml) or RNAse V1 (0.01, 0.02 or 0.05 U) was added and the reaction incubated for 1, 5 or 10 min (DMS), 20 mins (CMCT) or 5 min (RNase V1). The modified RNA was immediately ethanol precipitated on dry ice in the presence of 0.3 M ammonium acetate, washed with 70% ethanol and resuspended in 8 μl of water. The modifications were revealed by reverse transcriptase using ^32^P-labeled primers (for CSFV: 5′-CCTGTTAGTGGGCCTCTGCA-3′; for BDV: 5′-CCTATGCGTGGGCCTCTGCA-3′) and avian myeloblastosis virus reverse transcriptase (Promega). The products were resolved on a 7 M 6% polyacrylamide gel alongside RNA sequencing reactions on non-modified RNA similarly reverse transcribed, and were revealed using a phosphorimager (as above). RNA Selective 2′ Hydroxyl Acylation analysis by Primer Extension (SHAPE) analysis was conducted using 1-methyl-7-nitroisatoic anhydride (1M7) as a modifying agent and RNA secondary structures were modelled essentially as previously described (12,42,43). 6 pmol of RNA was resuspended in 18 μl of water, denatured at 80°C for 2 minutes and cooled down at room temperature for 10 minutes in buffer B (20 mM HEPES pH 7.5, 100 mM KOAc and 2.5 mM MgCl_2_). After 10 minutes incubation at 37°C RNAs were treated with 2 mM 1M7 or DMSO and incubated for 5 min at 37°C. The RNAs were cleaned by ethanol precipitation and pellets resuspended in 10 μl of water. Modifications were mapped by reverse transcription using 5′ fluorescent primers (D2 or D4 WellRED, Sigma Aldrich) and M-MLV RNAse H^−^ reverse transcriptase (Promega). For both BDV or CSFV IRES (WT or ΔIIId2) the following primer was used: Rluc: 5′ AACATTCATTTGTTTACATCTG 3′ complementary to the Renilla luciferase located downstream of the IRES. RNAs were denatured for 3 minutes at 95°C with 1 μl of DMSO and cooled in ice for 3 min. 3 μl of primer was added and samples were incubated for 5 min at 65°C and 10 min at 35°C and cooled on ice. Reverse transcription was performed in several steps: 2 min at 35°C, 30 min at 42°C and 5 min at 55°C. cDNA were separated by capillary electrophoresis (Beckman Coulter, Ceq8000). Data were analyzed using the software QuSHAPE (44). RNA probing was performed in triplicate for each IRES with distinct RNA preparations.

### BDV and CSFV infectivity assays

Full-length RNA transcripts corresponding to the wt and mutant BDV and CSFV genomes were produced *in vitro* using T7 RNA polymerase (Megascript kit, Ambion) as described by the manufacturer. The RNA transcripts were analysed for integrity by agarose gel electrophoresis and quantified using a Nanodrop spectrophotometer. CSFV RNA was electroporated into porcine PK-15 cells while BDV RNAs were introduced, in the same way, into ovine SFT-R cells. Following incubation for 72 h, the cells were stained with pan-pestivirus anti-NS3 antibodies (as described previously, (34)) while cell nuclei were stained with 4′,6-diamidino-2-phenylindole (DAPI) and images were captured using an epifluorescence microscope. Cell supernatants were harvested for virus titration in PK-15 cells and SFT-R cells. Viral titres were determined as previously described (31).

### SVV infectivity and growth curve assays

The wt and mutated pNTX-09-Age plasmids were linearised by digestion with *Swa I* immediately downstream of the virus poly-A tail. RNA was transcribed *in vitro* from the T7 promoter using an RNA transcription kit (Megascript; Ambion) according to the manufacturer's instructions and purified by phenol-chloroform extraction and ammonium acetate precipitation. Transcript quality was analysed by agarose gel electrophoresis. RNA transfection was achieved using 70–80% confluent 35 mm dishes of 293 cells with 1 μg of RNA and FuGENE HD transfection reagent (Promega) according to the manufacturer's instructions and harvested at appropriate times after transfection. For virus rescue assays, cells and medium were harvested at 72 h post-transfection and subjected to one round of freeze-thawing before being clarified at low speed and stored at –80°C for subsequent plaque or TCID_50_ assays. For quantitative RT-PCR assays, cells were harvested at 6 or 48 h post- transfection and stored at -80°C for subsequent RNA extraction.

For plaque assays, 6-well plates were pre-treated with 0.1% Poly-l-Lysine (Sigma) according to the manufacturer's instructions, seeded with 6 × 10^6^ 293 cells per well and incubated at 37°C overnight. Samples were serially diluted in 293 growth medium and 400 μl of each was plated out in duplicate. The wells were incubated at 37°C for 60 min and the inoculum removed and replaced with overlay (1% cell culture grade agarose [Sigma] in 293 growth medium). The plates were incubated at 37°C for 42 h and virus plaques revealed using neutral red stain.

For TCID_50_ assays, 96-well plates were seeded with 100 μl per well of 2 × 10^5^ 293 cells/ml. Virus samples were serially diluted in 293 growth medium and 50 μl added to the cells in each well (six wells were inoculated per dilution). The cells and virus were mixed by gentle agitation of the plates and incubated at 37°C for 24 h before the presence or absence of CPE was assessed for each well.

The titres of recovered viruses (wild-type and IIId2 deletion mutant) were determined after the third passage as TCID_50_/ml_._ 24-well plates were seeded with 1 ml per well of 2 × 10^5^ 293 cells/ml and immediately infected with rescued virus at a multiplicity of infection (MOI) of 0.01. At 3 h post-infection (h.p.i.) the inoculum was removed and replaced with 1 ml of 293 growth medium. The plates were incubated at 37°C. Wells were harvested at 3, 12, 24, 48 and 72 h.p.i. At each time point the cells and medium were harvested from the wells, subjected to one round of freeze-thawing, clarified at low speed and stored at –80°C for assay as TCID_50_.

### Quantitative RT-PCR

Total RNA was extracted from cells transfected with RNA derived from wild-type or the IIId2 deletion mutant using RNAzol-B (AMS Biotechnology) following the manufacturer's instructions. The level of viral RNA in each sample was determined by quantitative RT-PCR (RT-qPCR). Extracted RNA (1 μl) was added to a 50 μl RT-qPCR reaction mix (QuantiTect Probe RT-PCR kit; Qiagen) containing 400 nM of each primer (forward 5′-CTCGACCCTCCTTAGTAAGG-3′ and reverse 5′-GTTCTGCATATTTGTATGTGCTAC-3′) and 200 nM of a Taqman probe (5′-FAM-CGTGACTGCTACCACCATGAGTACATGG-TAMRA-3′) and cycled in an Applied Biosystems 7500 machine at 50°C for 30 min, 95°C for 15 min, 30 cycles of 94°C for 15s and 60°C for 60s. Fluorescence data was used to assign a cycle threshold value (C_t_) to each sample. The quantity of viral RNA in each sample was determined by reference to a standard curve obtained by RT-qPCR under the same conditions on a dilution series of transcribed viral RNA from the same genome region. Control experiments were performed in the absence of reverse transcriptase to ensure C_t_ values did not correspond to the input transfected plasmid DNA.

### Assembly of initiation complexes as determined by sucrose density gradient centrifugation

Initiation complexes were assembled on ^32^P-labelled IRES RNA transcripts. RNA (2 pmol in 10 μl) was denatured by heating to 80°C for 2 min and cooled to room temperature for 7 min in 20mM Tris pH 7.5, 2.5 mM MgCl_2_, 100 mM K acetate and 1 mM DTT. RRL (FlexiR-RRL, Promega) was pre-treated with either 4.8 mM cycloheximide or 10 mM GMP-PNP for 10 min at 30°C. RNA was incubated at 30°C for 20 min in 50 μl of pre-treated RRL in the presence of 0.5 mM Mg acetate, 75 mM K acetate, 10 μM amino acids and 8U RNasin (Promega). Reactions were stopped on ice, then were layered onto 15–50% sucrose gradients (25 mM Tris pH 7.5, 6 mM MgCl_2_, 75 mM KCl, 1 mM DTT) and centrifuged for 17 h at 24 000*g* at 4°C in a Beckman SW40 Ti rotor. Fractions (300 μl) were collected, one-third of which were vacuum blotted onto Hybond N+ membrane (Amersham), exposed and scanned using a phosphorimager. The amount of labelled RNA in each fraction was determined and expressed as the percentage of total counts.

## RESULTS

### BDV and CSFV sub-domains IIId2 are required for IRES activity

**Figure 1.**
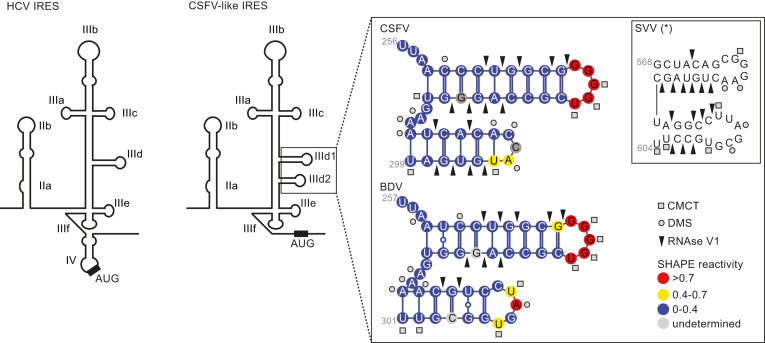
Schematic representation of the HCV (left) and CSFV-like (right) IRES structures. The names of the domains and sub-domains are indicated. The boxed area highlights the sub-domains IIId1 and IIId2 of BDV, CSFV and SVV IRES elements. The mean SHAPE reactivity from three independent experiments is indicated for each position on the BDV or CSFV IRESes fragment according to the color code on the figure. The chemical and enzymatic modifications achieved by DMS, CMCT and RNAse V1 are indicated using circles, squares and arrows, respectively. For comparison, modifications on the SVV IRES previously determined by Willcocks *et al* 2011 are shown (28).

We previously reported that SVV, a picornavirus, harbours an IRES element that is functionally related to the HCV IRES, capable of binding eIF3 and the 40S subunit, but is structurally closer to the CSFV IRES due to the presence of a bipartite IIId sub-domain (28). Furthermore, while the sub-domains IIId of HCV and IIId1 of CSFV are important for ribosome binding, the additional SVV IRES domain IIId2 is dispensable for the IRES activity (28). Before further characterizing the role of domain IIId2, we verified the formation of the predicted IIId2 stem–loop in the IRES elements of two pestiviruses, CSFV and BDV (26,45). To this end, we analysed the CSFV and BDV IRES structures in solution using Selective 2′ Hydroxyl Acylation analysis by primer extension (SHAPE), chemical and enzymatic probes. SHAPE analysis interrogates the RNA backbone flexibility at single-nucleotide resolution as flexible nucleotides can sample local conformations increasing the nucleophilic reactivity of 2′-hydroxyl groups toward 1M7. The sites of modification were then mapped as stops by primer extension reaction and capillary electrophoresis analysis, allowing us to assign quantitative SHAPE reactivity to individual nucleotides (Supplementary Figures S1 and S2). The SHAPE data was used in combination with accessibility information yielded by other chemical and enzymatic probes such as RNAse V1 reactivity, to detect double stranded regions, CMCT and DMS reactivity, to detect single stranded regions. Modification and cleavage sites were mapped by primer extension reactions and the reactivity assigned to individual nucleotides (Supplementary Figure S4). These combined data confirmed the presence of the expected IIId2 domains within the BDV and CSFV IRES elements (Figure 1 and Supplementary Figure S3). As shown in Figure 1, the CSFV sub-domain IIId2 is formed in solution and adopts the predicted stem-loop folding. Although double stranded restraints are less abundant within its stem, we now provide evidence that the BDV domain IIId2 is also folded in solution. These results confirm that both BDV and CSFV IRES elements contain the predicted sub-domains IIId2.

**Figure 2.**
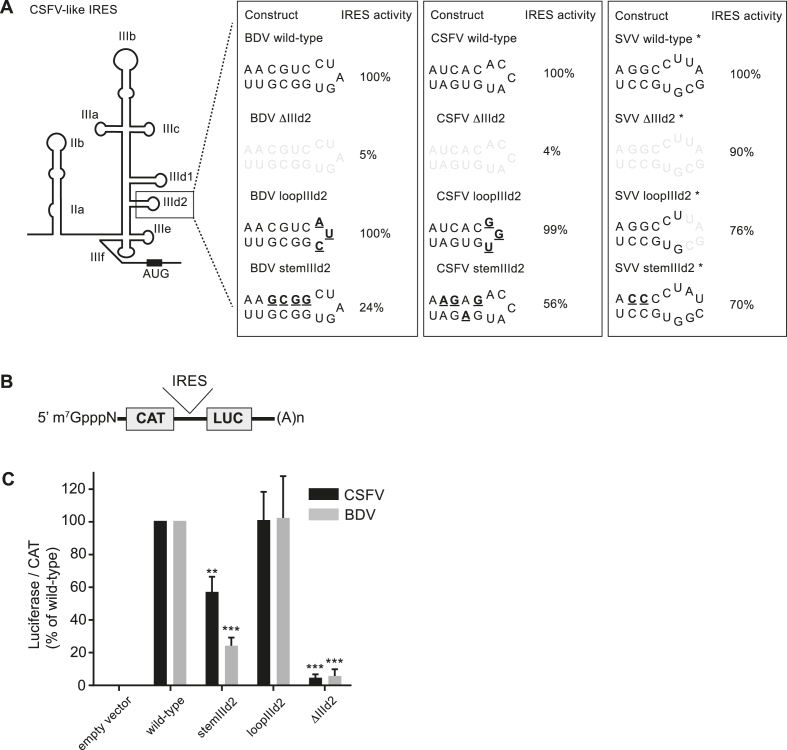
Effect of sub-domain IIId2 modifications on IRES activity. (**A**) Schematic representation of the CSFV-like IRES structures and mutations introduced in domains IIId2. Shaded nucleotides were deleted while mutations are underlined in bold. IRES activity is reported for each IRES construct. For comparison SVV IRES activities previously determined by Willcocks *et al.* 2011 are reported (28). (**B**) Schematic representation of the bicistronic mRNAs tested. IRES cDNAs were cloned between the chloramphenicol acetyl transferase (CAT) and luciferase (LUC) ORFs. (**C**) BHK cells, previously infected with the vaccinia virus vTF7–3 (which expresses the T7 RNA polymerase (38)) were transfected with bicistronic reporter plasmids as indicated. CAT and LUC activities were measured at 20 h post transfection as described in the Material and Methods section. The relative LUC/CAT ratio was set to 100% for wt IRES elements. Values are the mean ± SD from three independent experiments and were analysed by one-way ANOVA with Dunnett's multiple comparison test: *P< 0.05, **P< 0.01, ***P< 0.001, ****P< 0.0001 (GraphPad Prism 7.0).

The contribution of sub-domains IIId2 to BDV and CSFV IRES activity was then assessed. Wild-type (wt) or mutated IRES cDNA sequences were inserted between the CAT and LUC open reading frames of a bicistronic pGEM-CAT/IRES/LUC reporter plasmid (Figure 2A and B). The resulting plasmids containing a T7 promoter were transfected into BHK cells, infected with the vaccinia virus vTF7–3 that expresses the T7 RNA polymerase, and the expression from both CAT and firefly luciferase cistrons was assayed (Figure 2C).

Mutations within the IIId2 loop had no noticeable impact on the activity of either of these pestivirus IRES elements; however, mutations that disrupt the IIId2 stems reduced the IRES activities to 24% and 56% of that of the wt sequences for BDV and CSFV, respectively. More strikingly, and in stark contrast to the SVV IRES, deleting sub-domain IIId2 severely impaired IRES activity of both the BDV and CSFV IRES elements, leaving only 5% and 4% of wt activity (Figure 2C). To ascertain that these effects are not due to large RNA folding re-arrangements following the deletion of sub-domain IIId2, we probed the overall structure of wt or IIId2-deleted BDV or CSFV IRESs using SHAPE. In both cases the reactivity profile of the IIId2-deleted constructs are almost identical to the wild-type, apart from the two to three nucleotides surrounding the base of sub-domain IIId1 (Supplementary Figures S1 and S2). Importantly, this does not change the predicted 2D structure as modelled from the SHAPE data (Supplementary Figure S3). Thus, while sub-domain IIId2 is dispensable for the SVV IRES activity, it is required for efficient BDV and CSFV IRES activity.

### Sub-domains IIId2 are required for BDV and CSFV infectivity

**Figure 3.**
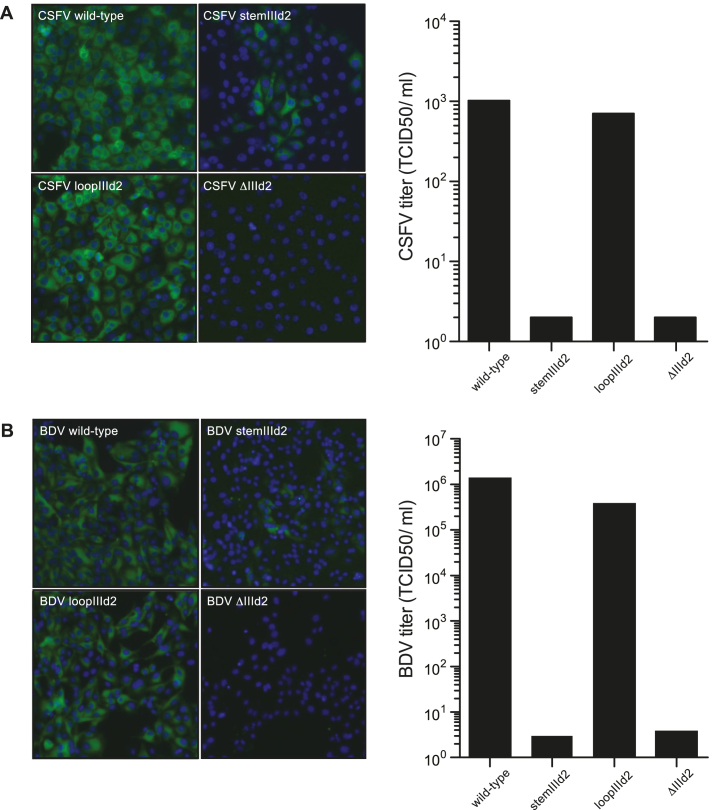
Deletion of the IRES sub-domain IIId2 from the pestiviruses BDV and CSFV impairs infection. (**A**) Full-length RNA transcripts derived from the wt CSFV Paderborn cDNA (pBeloPader10) and its indicated derivatives were introduced into PK-15 cells using electroporation. After 72 h, the cells were fixed and stained with antibodies against the pestivirus NS3 protein and the nuclei were visualized using DAPI. The viral titres in the cell supernatants prepared at 72 h were determined as TCID50/ml using virus titration in PK-15 cells. The data presented are representative of three independent biological replicates. (**B**) Full-length RNA transcripts derived from a wt BDV Gifhorn cDNA (in pBeloGif09.9.2) and its indicated derivatives were introduced into ovine SFT-R cells using electroporation. After 72 h, the cells were fixed and stained with antibodies against the NS3 protein and the nuclei were visualized using DAPI. The viral titres in the cell supernatants prepared at 72 h were determined as TCID50/ml using virus titration in SFT-R cells. The data presented are representative of three independent biological replicates.

Given the importance of the IIId2 domain for BDV and CSFV IRES activity, we next evaluated the effect of the various IRES mutations on BDV and CSFV replication. Mutations were introduced into a full-length cDNA of CSFV strain Paderborn (33) by targeted recombination; both wt and mutant RNA transcripts were generated *in vitro* and electroporated into PK15 cells as described before (31,34). Similarly, the corresponding mutations were also introduced into a full-length cDNA of BDV strain Gifhorn (derived from a BDV full length cDNA described by (33); Fahnøe *et al.*, in preparation). Full-length RNA transcripts prepared *in vitro* from the wt and mutant cDNAs were then electroporated into ovine SFT-R cells as described elsewhere (34). Virus infectivity was then assessed using immunofluorescence staining using pan-pestivirus anti-NS3 monoclonal antibodies to detect the CSFV and BDV NS3 proteins at 72 h post electroporation, or by measuring viral titers using TCID_50_ assays. Consistent with the IRES activity data, as measured by the reporter gene assay, mutations in the IIId2 loop had little impact on CSFV or BDV infectivity, while altering the stem of this sub-domain greatly reduced viral replication (Figure 3A and B). Furthermore, again echoing the results for IRES activity, deleting the entire IIId2 sub-domain eliminated CSFV and BDV replication (Figure 3A and B). Overall, these results demonstrated that the IIId2 sub-domains are important for both IRES activity and infectivity of BDV and CSFV.

### The BDV and CSFV IIId2 sub-domains are not required for 40S and eIF3 recruitment

**Figure 4.**
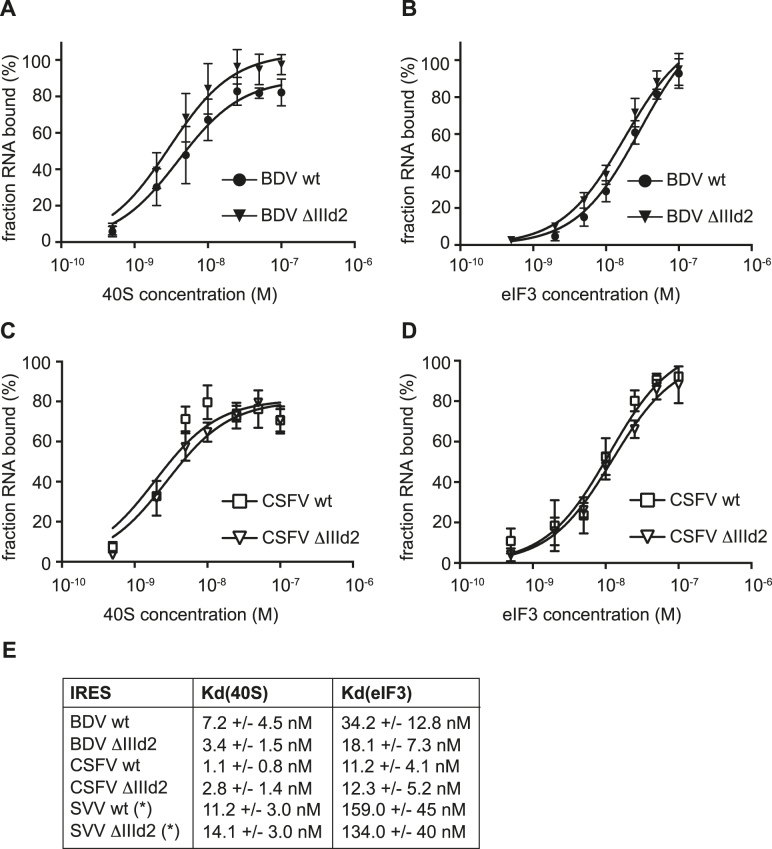
Deletion of sub-domains IIId2 does not affect the recruitment of 40S subunits and eIF3. (**A** and **B**) Binding curves of ^32^P-labeled wild-type (wt) or the ΔIIId2 mutant BDV IRES RNA transcripts to purified human 40S subunits or eIF3. Labeled RNAs were incubated with 40S subunits (A) or eIF3 (B) and binding assessed by filter binding assay. (**C** and **D**) Binding curves of ^32^P-labelled wt or ΔIIId2 CSFV IRES RNA transcripts to purified human 40S subunits or eIF3. Labelled RNAs were incubated with 40S subunits (C) or eIF3 (D) and binding assessed by filter binding assay. (**E**) Summary of dissociation constants (K_d_) calculated for 40S and eIF3 binding to wt and ΔIIId2 mutant IRES transcripts. For comparison K_d_ values for the SVV IRES previously determined by Willcocks *et al.* 2011 are shown (28). Indicated values are the average from a minimum of three repetitions with standard errors. All calculations were performed with GraphPad Prism 7.

To identify the role played by subdomain IIId2 in BDV and CSFV IRES-mediated translation, we assayed the interactions between RNA transcripts corresponding to the wt and mutant IRES elements and the translation machinery. Previous studies have shown that the first event in HCV-like IRES-mediated translation is the recruitment of the 40S subunit, followed by eIF3 binding and assembly of 48S complexes to yield elongation-competent 80S ribosomes (22). Therefore, eIF3 and ribosomal subunits were purified from HeLa cells according to described procedures (39). Then, individual purified components were incubated with wt or IIId2-deleted (ΔIIId2) ^32^P-labeled IRES transcripts, before conducting filter binding assays to analyze the affinity and specificity of the interactions (10). Given the importance of the HCV sub-domain IIId and CSFV sub-domain IIId1 in mediating 40S binding through base pairing with the 18S rRNA, we first tested the effect of IIId2 deletion on 40S recruitment (14–16,19–21,35,46). Both the wt BDV and CSFV IRES elements bind the 40S subunit with high affinity, with apparent equilibrium dissociation constants (*K*_d_) of 7.2 nM and 1.1 nM for BDV and CSFV, respectively (Figure 4A and C). The deletion of the entire IIId2 stem–loop had little impact on the binding affinity, with the calculated *K*_d_ remaining in a similar range, with values of 3.4 and 2.8 nM for the ΔIIId2 mutants of the BDV and CSFV IRES elements, respectively (Figure 4A and C). Similarly, the deletion of sub-domain IIId2 had little impact on eIF3 binding, with a *K*_d_ of 12.3 nM for the ΔIIId2 mutant against 11.2 nM for the wt and 18.1 nM (for ΔIIId2) against 34.2 nM (wt) for the CSFV and BDV IRES elements, respectively (Figure 4B and D). Of note, despite their structural similarities, the BDV and CSFV IRES elements have a higher affinity for eIF3 than that previously reported for the SVV IRES, which could be related to fine differences in the structure of the apical part of domain III (28). Overall, these results indicated that although sub-domain IIId2 is important for BDV and CSFV IRES activity, it does not influence 40S or eIF3 recruitment.

### The IIId2 sub-domains of BDV and CSFV, but not SVV, are important for 80S complex formation

The absence of an effect of deleting sub-domain IIId2 on 40S or eIF3 recruitment raised the possibility that this sub-domain plays a role at a later stage during translation initiation. Therefore, to understand better the role of sub-domain IIId2 in IRES-mediated translation initiation, the assembly of BDV, CSFV and SVV IRES RNA transcripts into translation initiation complexes *in vitro* was monitored using sucrose density gradient centrifugation. ^32^P-labelled wt and IIId2-deleted (ΔIIId2) IRES RNA transcripts were incubated with GMP-PNP- or cycloheximide (CHX)-treated rabbit reticulocytes lysate (RRL), to stall 48S and 80S ribosomal complexes, respectively. The assembly of the initiation complexes was then analyzed under identical conditions.

**Figure 5.**
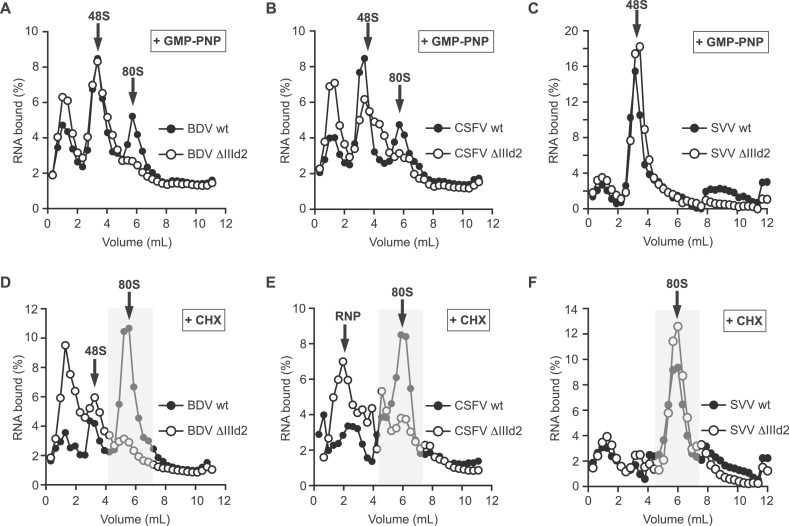
Assembly of ribosomal complexes on wt and mutant IRES transcripts as determined by centrifugation through 15–50% linear sucrose density gradients. Fractionation of initiation complexes formed after incubation of BDV (**A** and **D**) wt or ΔIIId2 IRES RNA transcripts, CSFV (**B** and **E**) wt or ΔIIId2 IRES transcripts and SVV (**C** and **F**) wt or ΔIIId2 IRES transcripts with RRL pre-treated with either GMP-PNP (A-C) or cycloheximide (CHX) (D–F). Arrows indicate the position of 48S complexes and 80S ribosomes. The profiles shown are representative of three independent experiments.

Incubation of all the wt IRES transcripts in GMP-PNP-treated RRL resulted mainly in the accumulation of 48S complexes, which migrate at about 3 ml from the top of the gradient (Figure 5A, B and C). In CHX-treated RRL, the assembly of 80S complexes was observed; these migrate at around 6 ml from the top of the gradient (Figure 5D–F). The peaks with low mobility at the top of the gradient represent RNPs assembled onto the RNAs. Next, we assessed whether the deletion of the IIId2 sub-domain affected the formation of stable initiation complexes. In GMP-PNP-treated RRL, the assembly of 48S complexes onto the ΔIIId2 BDV, CSFV and SVV IRES transcripts was not altered (Figure 5A–C). Similarly, in CHX-treated RRL, the assembly of 80S ribosomes onto the SVV IRES was not affected by the IIId2 sub-domain deletion, consistent with the observations that sub-domain IIId2 plays no major role in SVV IRES-mediated translation initiation (Figure 5F). In contrast, the assembly of 80S ribosomes onto ΔIIId2 BDV and CSFV IRES transcripts in CHX-treated RRL was strongly impaired and mainly resulted in the accumulation of RNP complexes (Figure 5D and E); it also appeared that the formation of slowly migrating RNA-protein complexes was enhanced on these mutant transcripts. Interestingly, in GMP-PNP-treated RRL, a small fraction of complexes can still progress to assemble 80S ribosomes onto wt BDV and CSFV IRES elements (Figure 5A and B), consistent with the previously described eIF2-independent initiation mechanism (47,48). However, the assembly of these complexes is also prevented by the sub-domain IIId2 deletion (Figure 5A and B). Therefore, these results strongly suggest that the pestivirus IRES sub-domain IIId2 coordinates the assembly of 80S ribosomes, while it has no effect on their assembly on the SVV IRES.

### Deletion of domain IIId2 impairs SVV infection

**Figure 6.**
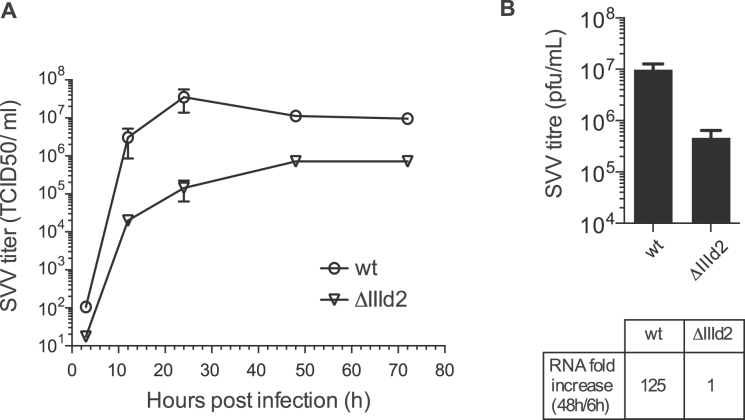
Effect of IRES sub-domain IIId2 deletion on SVV infectivity. (**A**) Growth characteristics of wt and ΔIIId2 SVV in cells. The growth of the SVVs in human 293 cells was evaluated using multi-step growth curves. Virus titres were determined as TCID_50_ /ml from harvests prepared at 3, 12, 24, 48 and 72 h post-infection. (**B**) Viral titres from 293 cells transfected with wt and ΔIIId2 SVV full-length transcripts were determined by plaque assay at 72 h post-transfection. RNA accumulation was evaluated by measuring the increase in viral RNA from 6 to 48 h post-transfection using quantitative RT-PCR. Values are the mean ± SD from three independent experiments.

Given that the presence of sub-domain IIId2 in the SVV IRES has no impact on IRES activity, unlike in the pestivirus IRES elements, we next hypothesized that the presence of this conserved domain could be important for some other aspect of SVV replication. To test this, the full-length infectious SVV-001 construct pNTX-09 was modified and used to generate mutant RNA transcripts lacking the IRES sub-domain IIId2 (36). These transcripts were introduced into human 293 cells by transfection. Infectious virus was rescued and the maintenance of the mutations within the rescued viruses was verified by RT-PCR and sequencing after three passages (data not shown). To examine the growth characteristics of the mutant viruses, 293 cells were infected with third-passage viruses at an MOI of 0.01 and, following incubation for 3, 12, 24, 48 and 72 h the virus yield was determined as TCID_50_. Figure 6A shows that the ΔIIId2 mutant SVV replicated significantly less well than the wt SVV, with a TCID_50_ titre about 100-fold less at each time point. To confirm these results, we also determined the infectivity of the wt and ΔIIId2 SVV RNA transcripts in cells. The transcripts were introduced into 293 cells and the cells were incubated for 72h before harvesting. The virus yield was then determined by plaque assay. RNA was also isolated at 6 and 48h and the level of viral RNA was measured by RT-qPCR. The results showed that the ΔIIId2 SVV titre at 72 h post-transfection was lower than the wt SVV, 5.5 × 10^+5^ versus 10^+7^ pfu/ml (Figure 6B). Furthermore, viral RNA accumulation from 6 to 48 h is >100-fold lower when sub-domain IIId2 is deleted (Figure 6B and Supplementary Figure S5). Overall, these results suggest that while sub-domain IIId2 has no role in SVV IRES activity, it is important for SVV infectivity.

## DISCUSSION

### Sub-domain IIId2 is important for IRES activity of pestivirus IRES elements and plays a role in both eIF2 dependent and independent pathways

HCV-like IRESs adopt a conserved architecture of ∼300–350nt, organized into domains II, III and, in some cases, IV, that drives multiple interactions with the cellular translation machinery (4–6). Such IRESs are found in a number of medically and economically important pathogens of humans and animals and have previously helped unravel new fundamental mechanisms of eukaryotic translation initiation. A number of structural and biochemical studies have highlighted the multiple roles that some of these individual domains and sub-domains play in interacting with the translational machinery to mediate IRES-dependent translation initiation, and therefore viral protein synthesis (7).

Among these conserved domains, domain II forms an L-shaped structure that binds across the 40S subunit interface to induce the conformational changes required to allow the assembly of elongation competent 80S ribosomes (16,18,25,47,49,50). Domain III binds in an elongated form at the back of the 40S subunits. Within domain III, sub-domains IIIb and the IIIabc junction are responsible for recruiting eIF3 while IIId, IIIe and IIIf establish direct high affinity contacts with the 40S subunit to ensure proper positioning of the start codon in the mRNA channel (16,18,49). Of particular importance, the apical loop of domain IIId establishes direct base pairing with the 18S rRNA ES7 helix 26 segment to form a binding interface where IIId and IIIe loops clamp onto the 18S ES7 segment (18,19). Moreover, this interaction is critical for locking the 40S ribosome into a conformation suitable for the 60S subunit joining (19).

Interestingly, some HCV-like IRESs found in pestiviruses and picornaviruses contain an additional sub-domain, IIId2, in the vicinity of the crucial IIId1 domain. In this study, we used a combination of *in vitro* translation assays, RNA-protein binding assays and virological assays to explore the role played by this conserved RNA domain, of yet unknown function.

Our results show that the CSFV and BDV pestivirus IRESs contain IIId2 sub-domains, and that these are important for both IRES activity and virus replication. While alteration of the IIId2 loop had no impact on IRES activity and viral replication, mutations destabilizing the IIId2 stem or the complete deletion of IIId2 impaired or abolished both IRES activity and viral replication. To further understand the molecular basis of these observations and establish the role of the CSFV and BDV IRES sub-domain IIId2 in IRES activity, we further analysed the interaction of these IRESs with the components of the translation machinery. Specific interactions of the CSFV IRES with 40S subunits and eIFs enable the assembly of preinitiation complexes containing eIF3 and ternary complex to form 48S complexes (25,47). By conducting filter binding assays, we established that CSFV and BDV sub-domains IIId2 are not required either for the recruitment of eIF3 or for the recruitment of 40S ribosomal subunits. The assembly of 48S complexes and 80S ribosomes was then assessed using sucrose density gradient centrifugation. While the deletion of CSFV and BDV sub-domain IIId2 did not impair the accumulation of 48S complexes in GMP-PMP-treated RRL, it dramatically reduced the assembly of 80S ribosomes in CHX-treated RRL. Moreover, and consistent with previous observations, we noticed that the conversion of 48S complexes, assembled on wild-type CSFV and BDV IRESs, into 80S ribosomes is only partially blocked by the presence of the non-hydrolysable analogue of GTP, GMP-PNP, thereby indicating partial independence of 80S assembly on GTP hydrolysis. In addition to the conventional eIF2-dependent mechanism of ribosome assembly, HCV-like IRESs can direct translation in the absence of eIF2 and its GTPase activity (47,48). This eIF2-independent pathway may be favoured in conditions of stress when cellular translation is inhibited, and eIF2 inactivated by phosphorylation, and is reminiscent of a bacterial mode of initiation whereby tRNA delivery and 80S assembly is driven by eIF3 and eIF5b, the eukaryotic counterpart of IF2, or other specialized factors such as eIF2A (48,51). Given that the deletion of sub-domain IIId2 resulted in the accumulation of just 48S complexes in GMP-PNP-treated RRL, we concluded that this sub-domain is important for the assembly of 80S ribosomes via both eIF2-dependent and independent pathways.

Interestingly, within the previously solved model of a ΔII–CSFV IRES–40S–eIF3–DHX29 complex, the domain III core structure consists of a rod-shaped scaffold bound to the back side of the 40S, from which sub-domains IIId1 and IIId2 protrude, and kinked at the hinge formed by the IIIabc junction to allow sub-domain IIIb to extend towards eIF3 (16). While the conserved motif GGG in the loop of sub-domain IIId1 is the major determinant of ribosome binding and initiation activity for all HCV-like IRESs, through establishing direct contacts with the ribosome, sub-domain IIId2 clearly extends away from the 40S subunit surface (16). Therefore, sub-domain IIId2 could be available and involved in direct interactions with eIF5b and/or the 60S subunit, thus explaining its role in both eIF2-dependent and independent 80S ribosome assembly pathways, and this is the subject of our ongoing studies. In addition, it was also recently proposed that eIF1A could play a role in HCV-driven IRES translation (52). In this revised model, it is suggested that eIF1A binding to the ribosome is stabilised by HCV-like IRESs that help to maintain the ribosome mRNA channel into a closed-like post-scanning conformation. This promotes the binding of the tRNA, in the absence or presence of eIF2, to stimulate 80S assembly, and we cannot exclude the possibility that sub-domain IIId2 could, at least in part, contribute to this mechanism.

### Sub-domain IIId2 is required for replication of the picornavirus SVV, but not for SVV IRES activity

In contrast to our observations that pestivirus IRES sub-domain IIId2 mediates the assembly of 80S ribosomes, this study and our previous data support a model in which the picornavirus SVV sub-domain IIId2 is not required for IRES activity. Our results clearly show that eIF3 and 40S recruitment, 48S complexes and 80S ribosomes assembly are all independent from the presence of sub-domain IIId2 (28). However, deletion of sub-domain IIId2 resulted in a 2-log reduction in both viral growth and viral titre throughout a 72 h infection. These results suggest that sub-domain IIId2 is not important for SVV IRES activity, unlike in pestivirus HCV-like IRESs, but still plays a role in viral infectivity.

Interestingly, several sub-domains of HCV-like IRESs can establish long-range RNA-RNA interactions that drive the progression of the infectious cycle (53). Some of these interactions are important for IRES activity and occur within the 5′UTR (54–59). Interactions involving HCV-like IRESs have also been reported between the 5′ and 3′ ends of the genome (60). The HCV IRES sub-domain IIId1 interacts with the 5BSL3.2 domain near the 3′ end of the genome and this interaction has been proposed to have a key role in the viral replication cycle by impairing IRES activity and promoting the switch from viral protein synthesis to RNA replication (61–65). Moreover, recent studies have suggested the existence of a much larger number of long range interactions than previously thought, as well as the importance of conserved structures in the coding regions (66,67).

High-throughput structural analyses based on SHAPE and molecular interference technologies have suggested that conserved nts within the IRES junctions IIIabc, IIIef and the PK2 sub-domains are key to controlling the activity of the dimer linkage sequence (DLS) and viral genome dimerization (60). It is proposed that these elements within the IRES act as chaperone-like partners to fine-tune the structure of 3′UTR motifs to control RNA replication. This circularization of the plus-strand genome could play an important role in the initiation of minus-strand RNA synthesis and/or regulation of a switch from RNA translation to genome replication (61–65). Therefore, it is tempting to speculate that, in the case of the SVV IIId2 sub-domain, this RNA element could be involved in establishing such topologies, that could be essential for controlling multiple steps of the infectious cycle, but also potentially increasing the local concentration of protein factors or providing protection against exonucleases that may target the viral RNA for degradation.

It has previously been proposed that the 5′UTR of several distantly related RNA viruses, in particular members of the hepacivirus and pestivirus genera (in the *Flaviviridae*), and *Picornaviridae*, contain conserved structured IRES domains (26). Despite the fact that some peripheral sub-domains do differ from a proto-typical HCV-like IRES scaffold, most of the core elements such as domain II, domain IIId and IIIe and the pseudoknot are conserved (26). These findings suggested that IRESs in the 5′UTR of RNA viruses consist of modular elements that may have been exchanged between unrelated members of distinct and distantly related enveloped or non-enveloped RNA virus families by recombination. The exchange of RNA modules by recombination occurs commonly, contributing to the evolution of RNA viruses such as members of the picornavirus and pestivirus genera (68–70). Moreover, HCV-like IRESs can functionally replace picornaviruses IRESes within infectious picornaviruses (71).

In summary, our studies show that the IIId2 sub-domain is clearly important for IRES activity in the pestiviruses CSFV and BDV but for RNA replication in the picornavirus SVV. This supports the hypothesis that once exchanged, RNA domains, such as sub-domain IIId2, can evolve independently to acquire distinct functions in unrelated viruses and therefore has broader implications for the molecular evolution of viruses.

## Supplementary Material

Supplementary Data
