# Supplementary material for: Distinct roles for the IIId2 sub-domain in pestivirus and picornavirus internal ribosome entry sites
Source: Nucleic Acids Res. 2017 Oct 24;45(22):13016–28. doi: 10.1093/nar/gkx991 (PMC5727462; doi:10.1093/nar/gkx991)
Supplement: Supplementary Data [file gkx991_supp.pdf]

Supplementary Figure S1

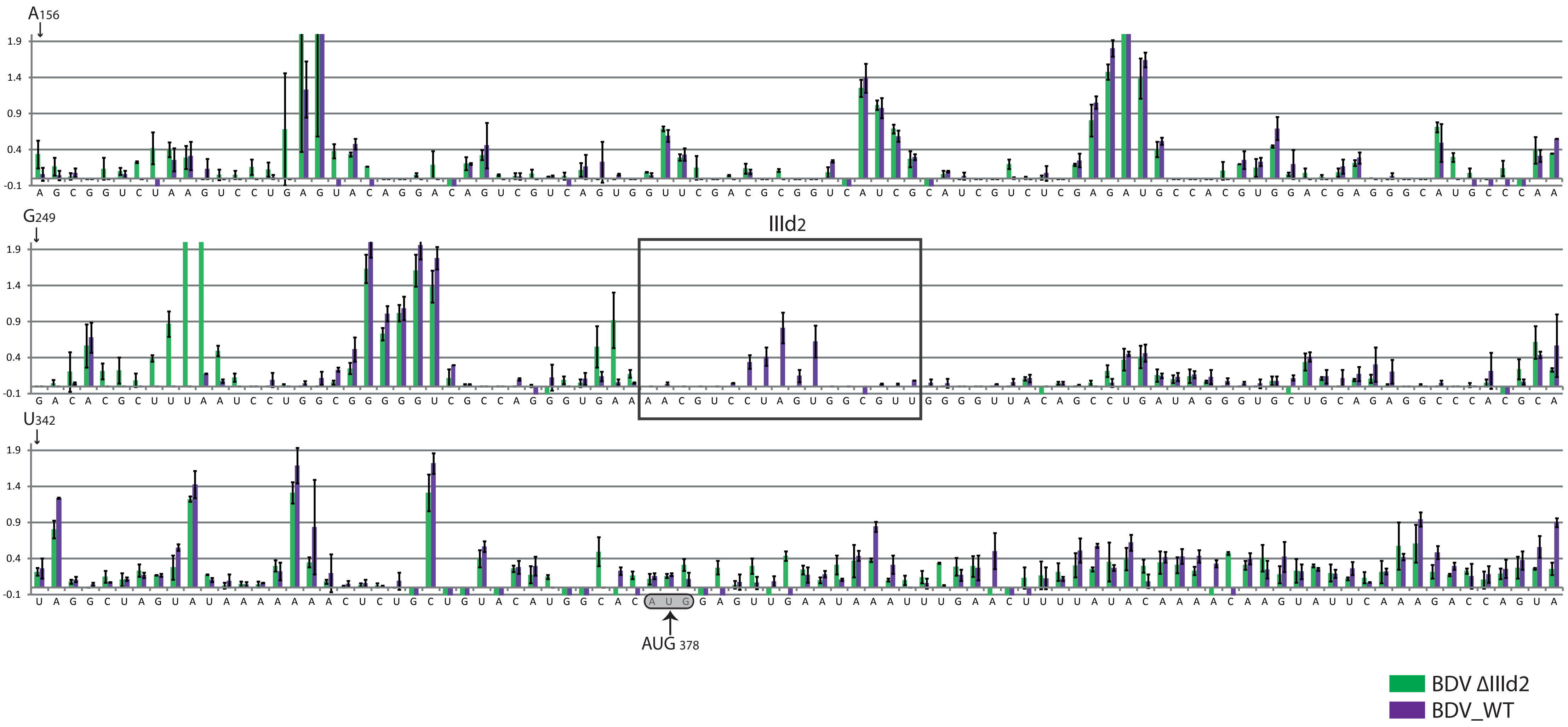

Supplementary Figure S1: Shape probing of the BDV IRES WT or BDV IRES ΔIIId2. Histogram show the reactivity value for 1M7 as calculated with QuSHAPE. WT BDV IRES reactivities are shown in purple while BDV IRES ΔIIId2 reactivities are in green. IIId2 nucleotides that were deleted in BDV IRES ΔIIId2 are boxed. Error bars represent the s.e.m of three independant experiments.

Supplementary Figure S2

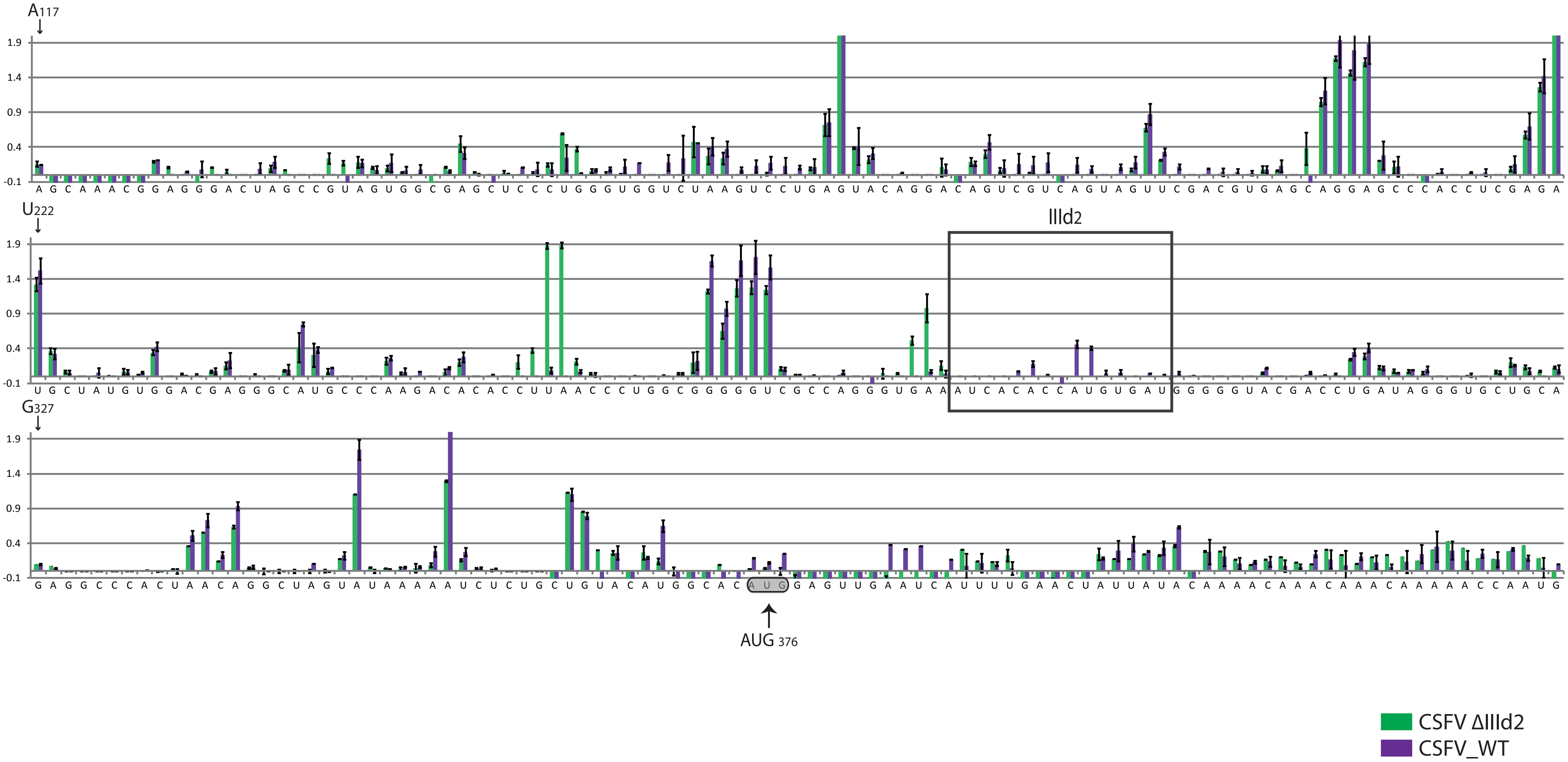

Supplementary Figure S2: Shape probing of the CSFV IRES WT or CSFV IRES ΔIIId2. Histogram show the reactivity value for 1M7 as calculated with QuSHAPE. WT CSFV IRES reactivities are shown in purple while CSFV IRES ΔIIId2 reactivities are in green. IIId2 nucleotides that were deleted in CSFV IRES ΔIIId2 are boxed. Error bars represent the s.e.m of three independant experiments.

Supplementary Figure S3

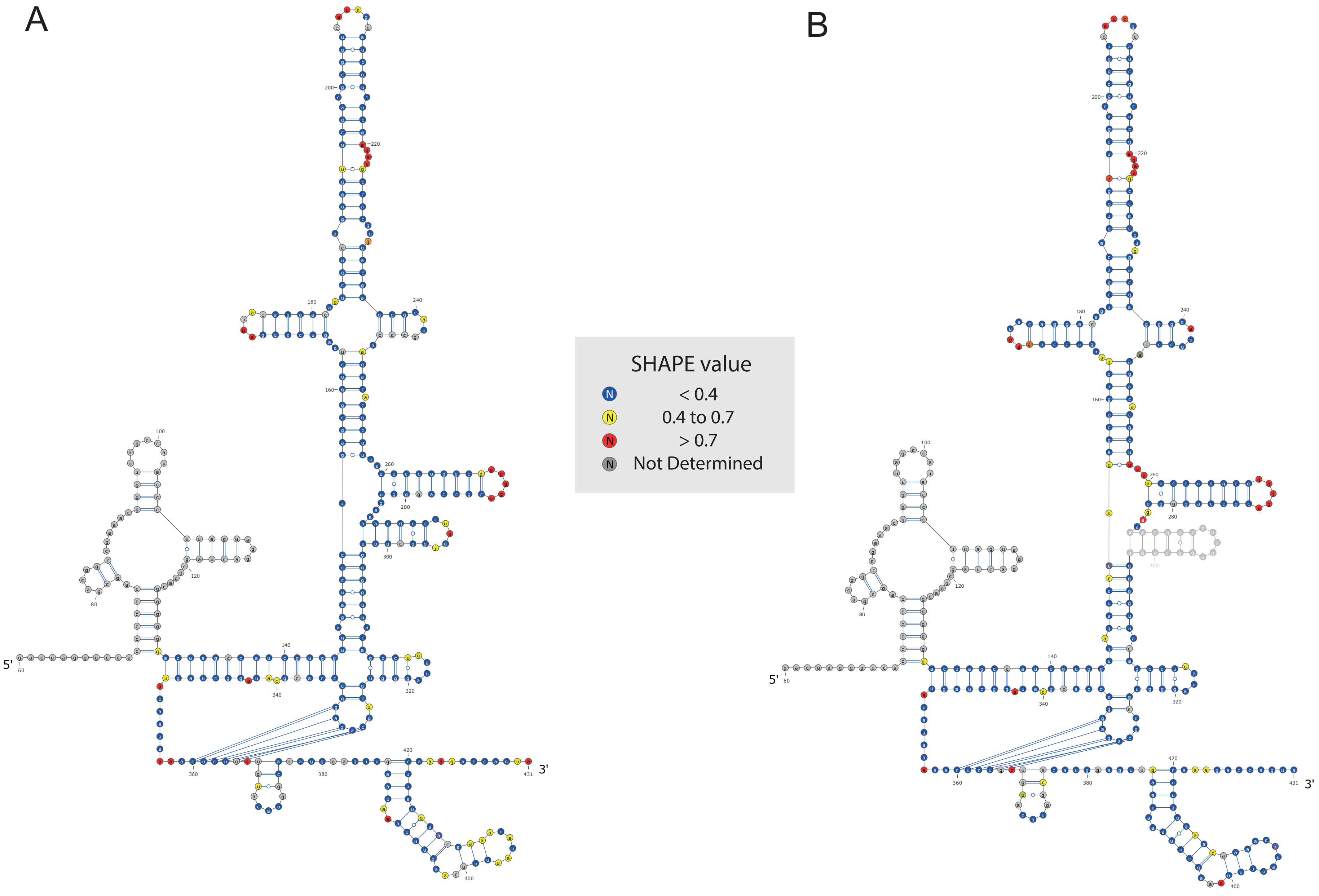

BDV wild-type

BDV ΔIIId2

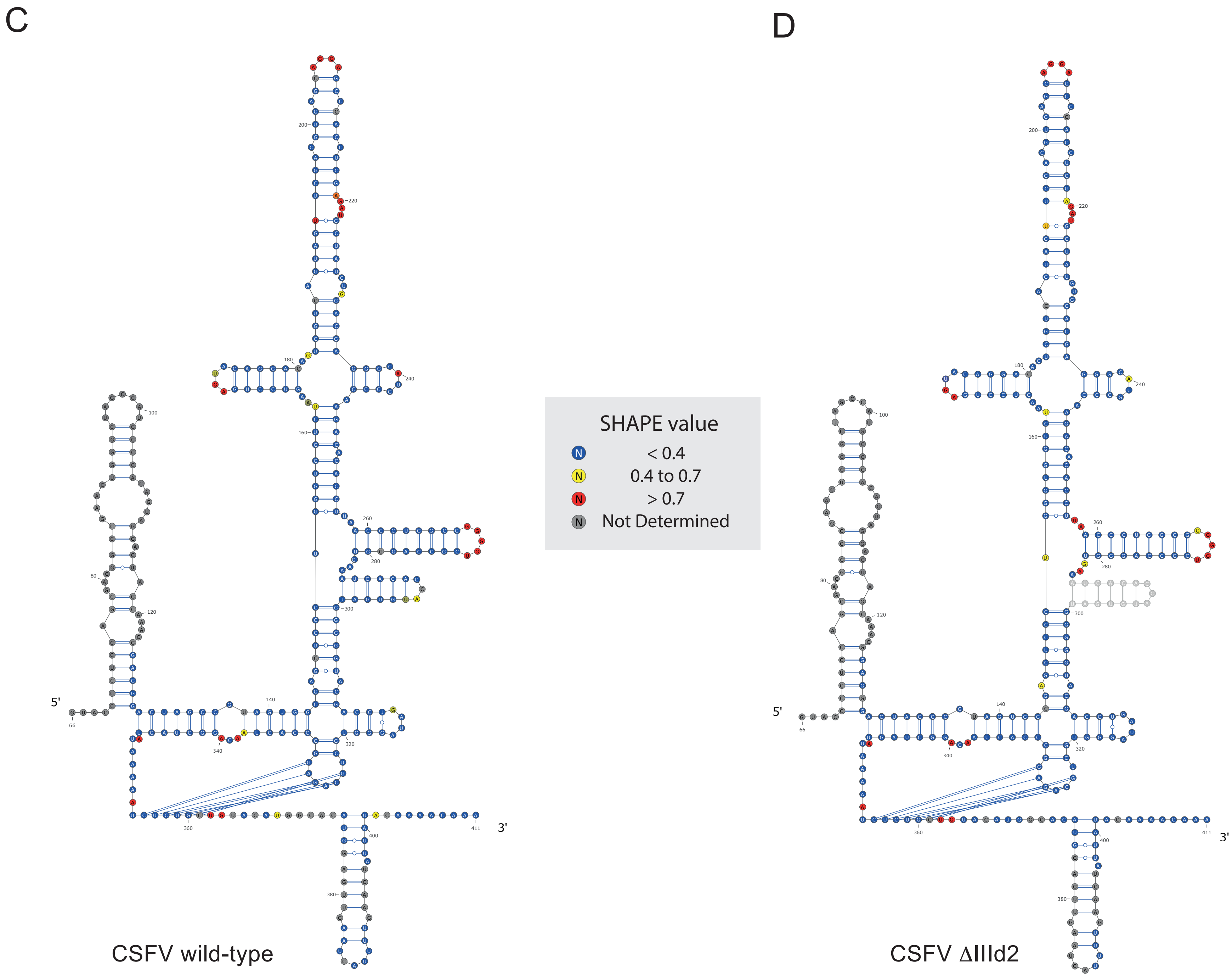

Supplementary Figure S3: Secondary structure model for BDV WT (A), BDV ΔdIII2 (B), CSFV WT (C) and CSFV ΔdIII2 (D). The shape reactivity is colour encoded as shown in the box.

Supplementary Figure S4

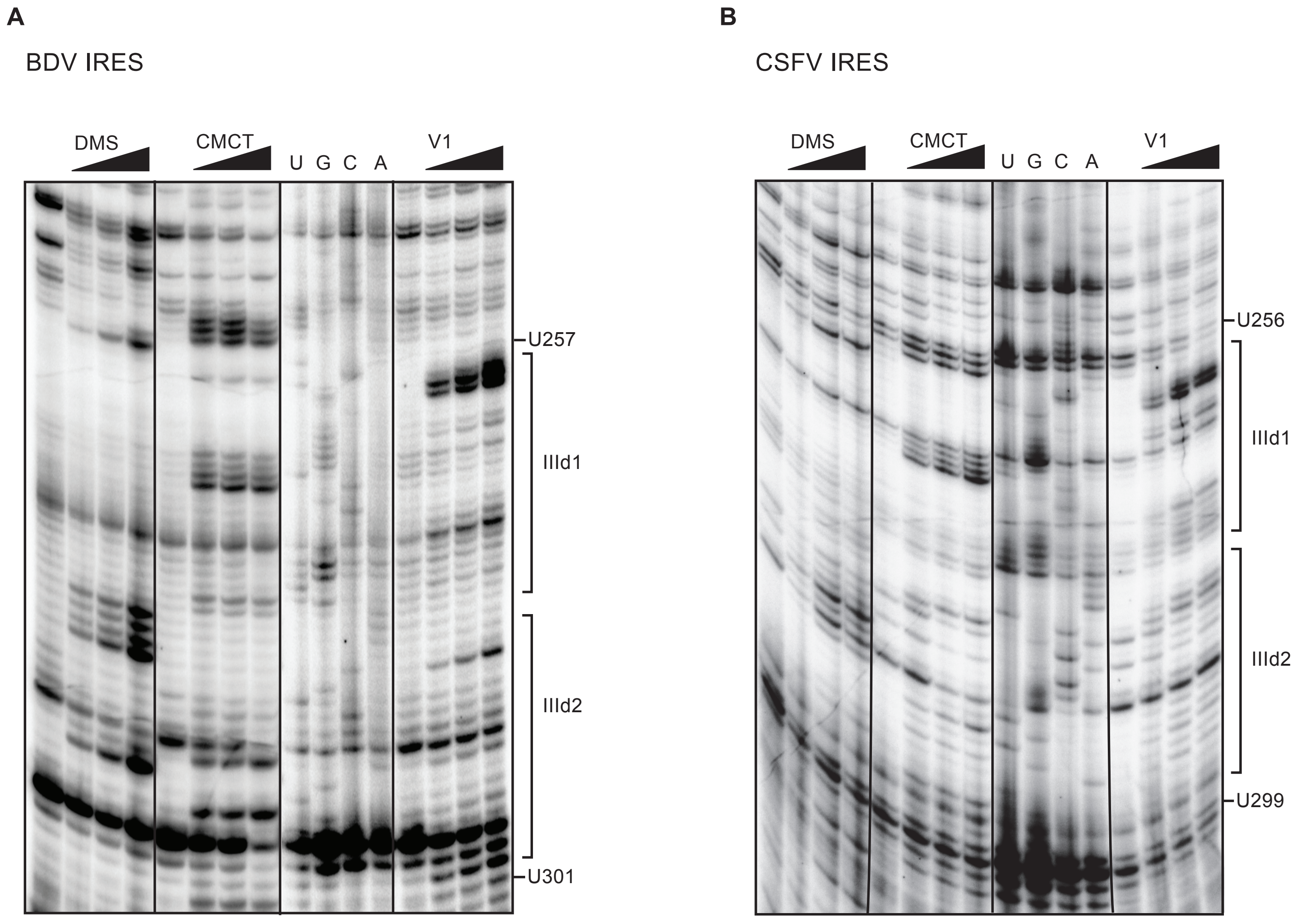

Supplementary Figure S4: Primer extension analysis of the BDV and CSFV IRESs RNA structure in solution. Primer extension analyses of the BDV (A) or CSFV (B) IRESs cleaved by RNase V1, DMS and CMCT. All reactions were performed in conditions described under “Material and Methods”. RNAs were treated with increasing amount of CMCT (2, 4 or 10 mg/ml) for 20 mins, increasing amount of RNAse V1 (0.01, 0.02 or 0.05 U) for 5 mins or with 0.395M DMS for 1, 5 or 10 mins. Lanes U, G, C, and A correspond to the sequencing ladders. Numbering of the nucleotides in the BDV/CSFV RNA is indicated on the right side of the panels. Positions of IIId1 and IIId2 subdomains are indicated on the right side of the panels and the gels shown are representative of at least three independent replicates.

Supplementary Figure S5

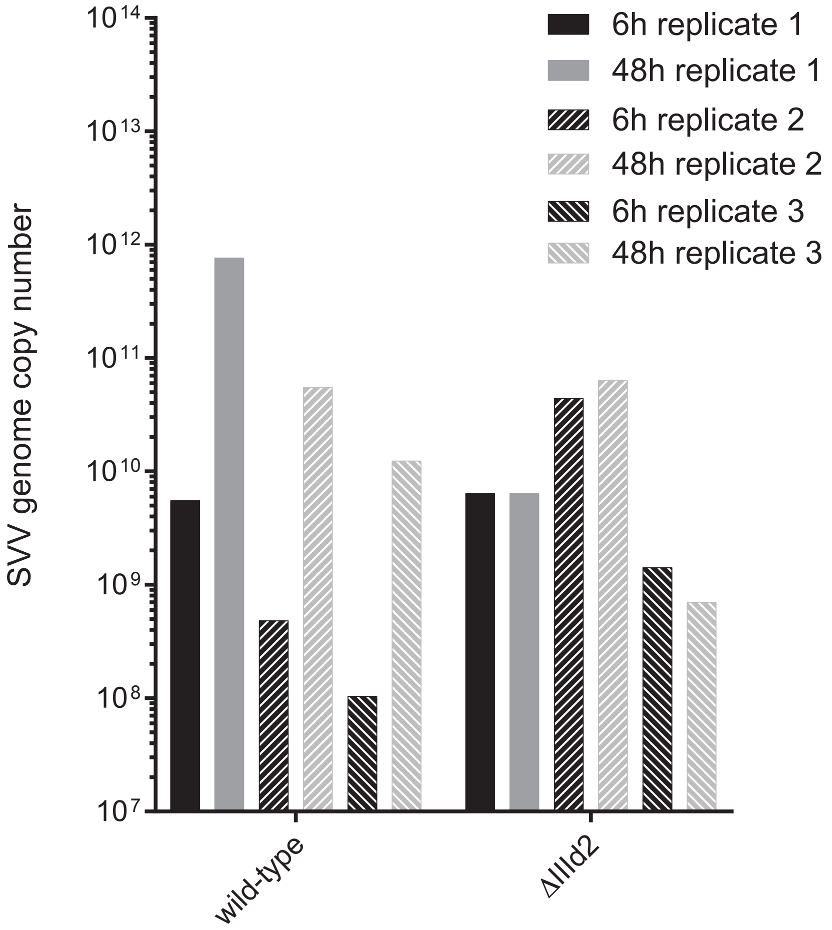

Supplementary Figure S5: Effect of IRES sub-domain IIId2 deletion on SVV RNA accumulation. 293 cells were transfected with wt and ΔIIId2 SVV full-length transcripts and RNA accumulation was evaluated by measuring the RNA from 6h to 48h post transfection using quantitative RT-PCR. Individual data obtained for 3 independent replicates are displayed separately to reflect the different initial amount transcripts transfected.
